# On-Demand Tailoring between Brittle and Ductile of Poly(methyl methacrylate) (PMMA) via High Temperature Stretching

**DOI:** 10.3390/polym14050985

**Published:** 2022-02-28

**Authors:** Changchun Wang, Jia Xi Pek, Hong Mei Chen, Wei Min Huang

**Affiliations:** 1 Jiangsu Key Laboratory of Advanced Structural Materials & Application Technology, School of Material Science and Engineering, Nanjing Institute of Technology, Nanjing 211167, China; 2 School of Mechanical and Aerospace Engineering, Nanyang Technological University, 50 Nanyang Avenue, Singapore 639798, Singapore; jpek008@e.ntu.edu.sg; 3 College of Chemistry and Materials Science, Sichuan Normal University, Chengdu 610066, China; chenhongmei@sicnu.edu.cn

**Keywords:** amorphous, poly(methyl methacrylate), brittle, ductile, hot-stretching, toughness, Young’s modulus, aging

## Abstract

**Dog-bone** shaped poly(methyl methacrylate) (PMMA) samples were pre-stretched at different temperatures (within the glass transition range and slightly above) to different strains. Subsequently, these pre-stretched samples were aged at 40 °C for up to three months, and finally, all samples were uniaxially stretched to fracture. The Young’s modulus, ultimate stress and toughness of the samples were obtained and plotted as a function of the temperature, and strain in pre-stretching in the contour format. The influence of aging was revealed when the contours of different aging times were compared. One of the most interesting findings was that the toughness of this PMMA can be tailored via controlling the temperature and strain in pre-stretching. The toughness of the pre-stretched samples ranged from 1.317 MJ/m^3^ to 23.281 MJ/m^3^ (without aging) and from 1.476 MJ/m^3^ to 27.532 MJ/m^3^ (after three months of aging). Based on the results of a series of additional experiments, a mechanism was proposed to reveal the fundaments behind the influence of the temperature and strain in pre-stretching and aging.

## 1. Introduction

Although it is possible to modify poly(methyl methacrylate) (PMMA) to form crystalline structures as recently reported in [1], commercial PMMA is mostly amorphous. Amorphous PMMA is one of the most widely used engineering polymers due to its low cost, high transparency, easy processing and polishing, and excellent UV tolerance. However, its low toughness, i.e., limited flexibility and poor impact resistance [2,3,4], prevents it from being applied in many engineering applications.

The physical properties of polymers, such as the Young’s modulus, yield stress/strain, fracture stress/strain and toughness, are strongly influenced by not only the internal structure, which may be modified by the thermo-mechanical treatment, but also the testing conditions, such as the environmental temperature, sample shape, type of load and loading rate [5]. The concept of physical aging was proposed several decades ago [6,7,8,9]. After thermo-mechanical processing, physical aging at around room temperature over a prolonged period, which is common in engineering practice, may induce significant changes in some mechanical properties of polymers [10,11,12,13]. Numerous experimental and theoretical works have been documented in the literature about the influence of physical aging of amorphous polymers [14,15,16,17]. Nevertheless, most of these studies are limited to small processing deformation.

For amorphous polymers, there are two conventional routes to improve their toughness: namely, incorporating a discrete rubbery phase via blending and copolymerization [18,19]; and adding a reinforcement [20,21]. However, high toughness and high strength tend to be mutually exclusive after such a modification [22]. Without any modification of the material itself, high temperature/high strain stretching (well above the glass transition temperature, T_g_) has been applied to convert brittle amorphous polymers, such as PMMA, into ductile polymers, if stretched again along the same direction of pre-stretching [23]. The investigation to reveal the actual underlying mechanism of this brittle-ductile transition is still in progress [24].

The shape memory effect (SME) refers to the capability of a severely and quasi-plastically deformed material to recover its original shape, but only when a right stimulus is applied [25]. According to [26], most polymers have the heating/chemo-responsive SME. Excellent heating responsive SME based on the glass transition has been reported in typical amorphous polymers, such as PMMA and polycarbonate (PC) [27,28].

For the heating-responsive SME in polymers based on the glass transition, the standard process of a full shape memory cycle includes two steps. In the first step, the polymer is heated to its glass transition temperature (T_g_) for softening and then deformed. After cooling and subsequent removal of the constraint, the deformed shape is mostly maintained. This step is technically called programming, and the deformed shape is normally called the temporary shape. For relatively ductile polymers, such as PC, programming can be done at room temperature. In the second step, the programmed polymer is heated to high temperatures (above its T_g_), so the polymer returns its original shape (also called permanent shape) from the temporary shape. As reported in [28], after aging at 40 °C for up to one year, the PMMA samples programmed via high temperature (within the glass transition range and slightly above) uniaxial stretching to up to 80% strain are still able to almost fully recover their original shape upon heating.

Above mentioned high temperature stretching for brittle-ductile transition is actually the first step of the shape memory cycle. For polymers with excellent SME (such as PMMA and PC [27,28]), after one complete shape memory cycle, not only the shape, but also other thermo-mechanical properties, are largely recovered.

As amorphous PMMA has been confirmed to be an excellent shape memory polymer (SMP), in this study, we investigate the influence of stretching temperature (programming temperature), stretching strain (programming strain) and aging (at 40 °C for up to three months) on the Young’s modulus, ultimate stress (the maximum engineering stress until fracture) and toughness of the programmed/pre-stretched PMMA samples. A series of additional tests are also carried out in order to reveal the underlying mechanism for the brittle-ductile transition.

## 2. Material, Sample Preparation and Experimental

### 2.1. Material and Samples Preparation

The same poly(methyl methacrylate) (PMMA) (from Ying Kwang Acrylic, Singapore) as in [29] was used in this study. Two types of dog-bone shaped samples were laser cut from 1 mm- and 2 mm-thick sheets. Refer to Figure S1 in Supplementary Materials for the dimensions of these two types of samples. Smaller sized samples (about 10 mg) were also prepared for differential scanning calorimetry (DSC) tests.

### 2.2. DSC Test

DSC tests were conducted using a DSC, TA Q200 instrument (TA Instrument, New Castle, DE, USA) using three types of samples. The first type was as received, and the second type was pre-heated at 200 °C for two hours to eliminate the influence from the previous material manufacturing process and then stored in an oven at 40 °C for two months—these samples were conducted at a heating/cooling rate of 5 °C min^−1^ between 20 °C and 240 °C for two cycles. The third type was also as received, and the DSC tests were carried for two cycles at the same heating/cooling rate as applied in the other two types. In each test of the third type, in the first cycle, the sample was heated from 20 °C to a prescribed temperature (120 °C, 150 °C, 165 °C or 185 °C) and then cooled back to 20 °C. In the subsequent second cycle, the sample was heated to 240 °C.

### 2.3. Pre-Stretching/Compression and Cyclic Uniaxial Tensile Tests

Unless otherwise stated, all dog-bone shaped samples (1 mm-thick, Type I. Refer to Figure S1 in Supplementary Materials for sample dimensions) were pre-annealed at 120 °C for two hours.

An Instron 5565 (with a hot chamber, from Instron, MA, USA) was used for uniaxial pre-stretching at a strain rate of 0.2%/s and a predetermined temperature (namely, 100 °C, 110 °C, 120 °C or 130 °C) to three different prescribed strains, namely 10%, 40% and 80%, respectively. In each test, the clamped sample was pre-heated in the hot chamber at the required temperature for half an hour for temperature stabilization before stretching. When the tested sample was stretched to the required strain, the heater was switched off and the door of the hot chamber was opened for cooling. When the sample was cooled back to the ambient temperature (about 23 °C), the stretched sample was unloaded and then taken out of the machine.

Some pre-stretched samples (1 mm, Type I) were kept in an oven at 40 °C for up to three months.

Cyclic uniaxial tensile tests were carried out at room temperature in a similar way as mentioned above, to 1% in the first cycle, 3% in the second cycle and then to fracture in the last cycle. In these cycling tests, unloading was stopped when the stress was about 2.5 MPa for the samples of Type II or about 0 MPa for the samples of Type I.

To investigate the influence of pre-treatment without storage (aging), the original samples and pre-heated samples (at 100 °C, 110 °C, 120 °C or 130 °C for half an hour), samples pre-stretched or pre-compressed at high temperatures and shape recovered samples were also tested. Pre-stretching was conducted in a similar way as mentioned above at 160 °C to 150% strain. The pre-compressed samples were compressed by 9% or 23% in the thickness direction at 160 °C. The shape recovered samples were pre-stretched (150% strain at 160 °C) and then heated at 160°C for shape recovery. Refer to [29] for the heating-responsive SME of this PMMA. The samples used here were Type II.

It was found that there is not much significant difference between the samples made of 1 mm or 2 mm thick sheets. Refer to Figures S2 and S3 in Supplementary Materials for their stress versus strain relationship in cyclic uniaxial tension.

Herein, the stress and the strain are meant for the engineering stress and engineering strain, respectively. They were worked out based on the measured dimensions (gauge length, width and thickness) of each sample before testing.

### 2.4. X-ray Diffraction (XRD) Test

For XRD test (Bruker D8 Advance, Massachusetts, Germany), the diffraction angle 2*θ* was from 5° to 90° with a scanning speed of 5° per minute. Copper was used as the source of X-ray radiation. Two original samples and one pre-stretched sample (to 150% strain at 160 °C) were tested.

### 2.5. Photoelasticity Test

Photoelasticity is a traditional technique to reveal the internal stress field in transparent polymers [30]. In [27], the internal stress field of programmed PC (via stretching), another typical engineering amorphous polymer, was captured using photoelasticity. In [31], this method was applied to monitor the evolution of the internal stress field in ethylene-vinyl acetate (EVA) during the shape memory cycle. In the course of this study, the tested samples were placed right in front of a computer monitor, which provided the polarized light source. Through a polarizer, the internal stress field of the examined samples was revealed.

## 3. Results and Analysis

### 3.1. DSC

**Figure 1:**
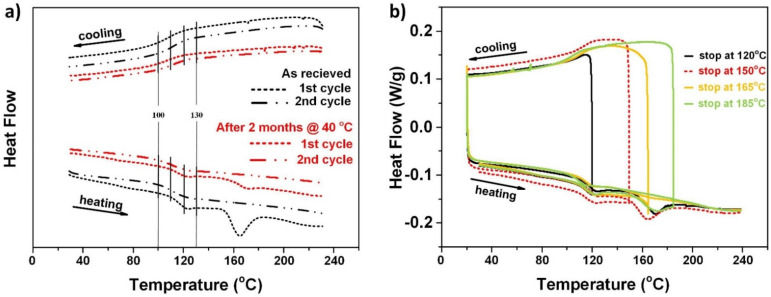
DSC results. (**a**) As-received and stored for two months after heat treatment (200 °C for two hours); (**b**) cyclic test.

In Figure 1a, we can see not only the glass transition, but also “melting” in the first heating process of the as-received sample. However, there is no apparent crystallization transition in the following cooling process, and the “melting” transition disappears in the second heating process. On the other hand, for the sample that has been pre-heated to 200 °C for two hours and then stored for two months at 40 °C, the same trend is observed, although the magnitude of the “melting” trough in the first heating process appears to be much less significant.

Figure 1b presents the results of four cyclic DSC tests (heated to different temperatures in the first heating process) to further investigate the “melting” transition. It appears that the significance of this “melting” transition in the second heating process highly depends on the highest temperature in the previous heating process. Pre-heating over the “melting” temperature range fully eliminates this “melting” transition in the subsequent heating process.

While after a period of storage at low temperatures (e.g., two months at 40 °C in the current study), the “melting” trough shows up again, and there is no apparent crystallization transition in the cooling process in all DSC tests. On the other hand, the glass transition temperature range in heating or cooling is always stable.

Although still debated [32,33,34,35,36], one possible explanation is that PMMA is a typical amorphous polymer, but there are local order regions in the matrix [37]. The local orientational order is associated with the local chain stretching, and is of the uniaxial nematic type [38]. The neighboring chain segments are packed more or less parallel to each other to form nodular bead-like structures. Several groups have reported such nodular structures in many amorphous polymers, such as polystyrene (PS), PC and polyethylene terephthalate (PET) [39,40,41]. The nodular beady structures have a tendency to aggregate and form “large” ordered regions, called “paracrystalline” [37]. The “melting” transition in the first heating process in this PMMA might be the result of the melting of the “paracrystals”. However, immediate cooling back to the room temperature is not sufficient for “paracrystals” to be reproduced, since the “paracrystals” only gradually form at low temperatures over a long time. Hence, no crystallization transition can be observed in the cooling process in above DSC results. As mentioned above, this is only one possible explanation, and further investigation is required.

This phenomenon may be used as the working mechanism for temperature sensors to monitor over-heating as reported in [42].

### 3.2. Cyclic Uniaxial Tension

Experimental results revealed that there is not much difference between the samples cut from 1 mm and 2 mm sheets. Hence, only the results of 2 mm samples (Type II) were reported here to reveal the influence of pre-treatment (pre-heating or pre-stretching at high temperatures) without storage, while the results of 1 mm samples (Type I) were reported here to reveal the influence of storage after high temperature stretching.

**Figure 2:**
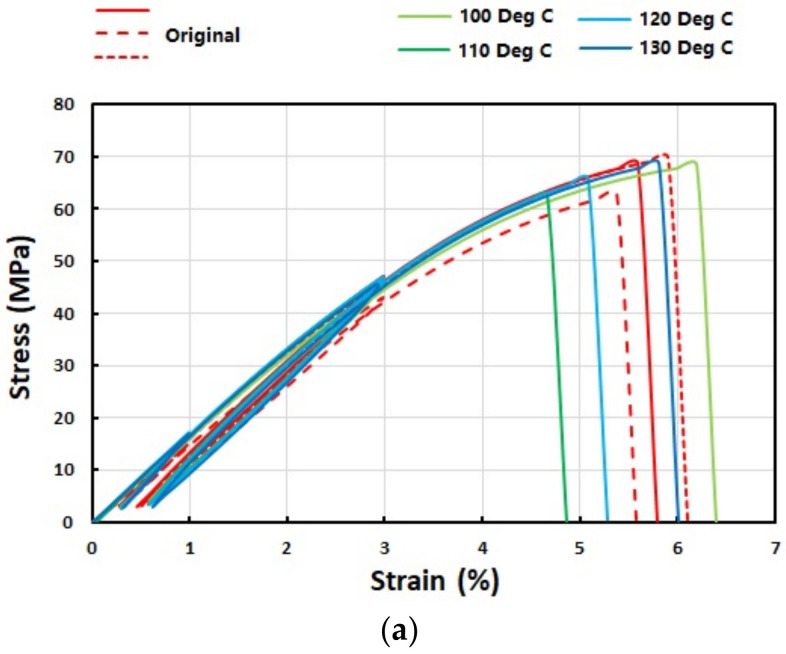

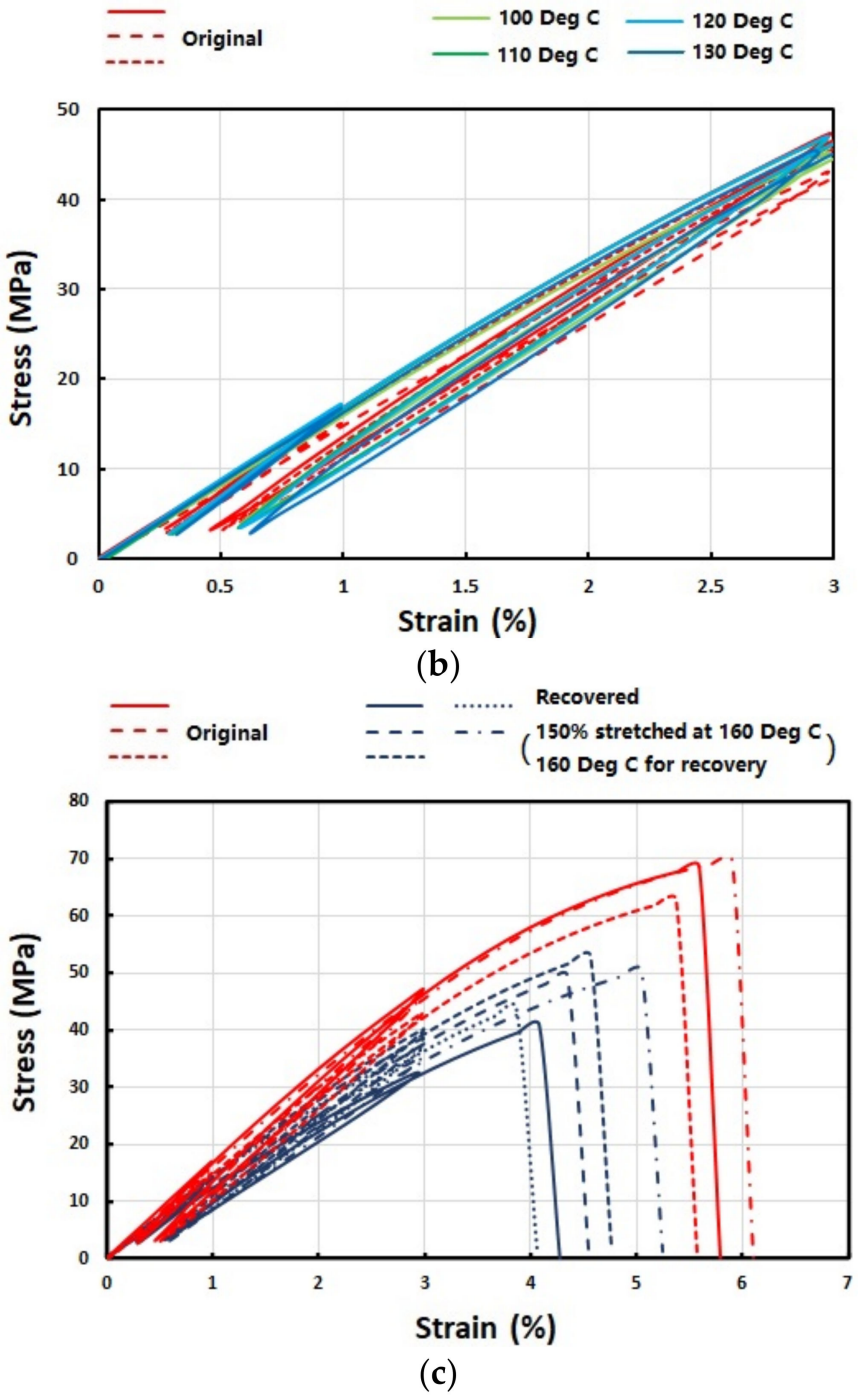
Cyclic uniaxial tension results of (**a**) original (three pieces) and preheated samples (to 100 °C, 110 °C, 120 °C and 130 °C, four samples); (**b**) zoom-in view in the small strain range (≤3%) of (**a**); and (**c**) original (three pieces) and recovered (five pieces) samples.

The stress versus strain relationships in cyclic uniaxial stretching of three original (as-received) samples and four pre-heated samples (all type II, without storage) are presented in Figure 2a. Figure 2b is the zoom-in view of their stress versus strain curves in the small strain range (≤3%). It is apparent that the fracture of all these samples is brittle and the effect of pre-heating is negligible. In Figure 2c, the results of five recovered samples are compared with those of three original samples. The recovered samples were pre-stretched to 150% strain at 160 °C, and then heated to 160 °C for shape recovery. Same as that reported in [29], the recovery was almost 100% in all these samples. It appears that the recovered samples are slightly softer, and with a slightly lower fracture stress and strain. At this point, we can conclude that, the same as the original samples, the pre-heated and recovered samples are still brittle.

**Figure 3:**
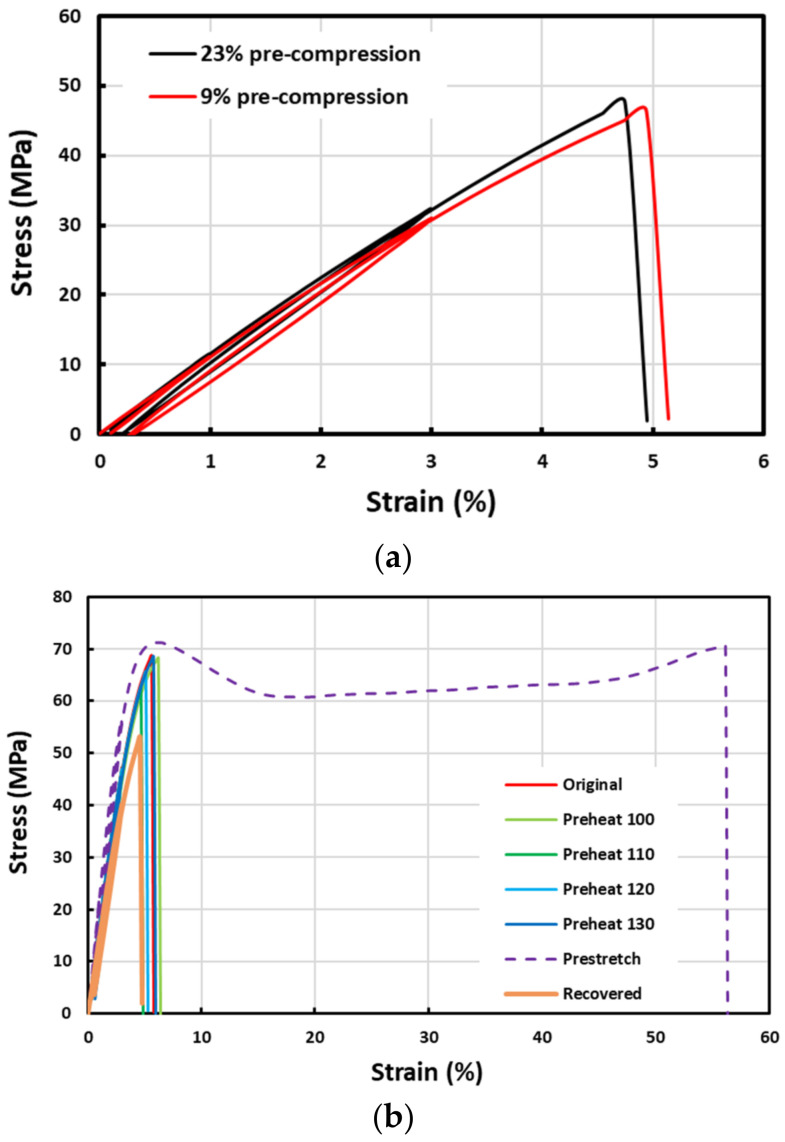
Stress versus strain relationships in cyclic uniaxial tension of (**a**) pre-compressed samples (two pieces with 9% and 23% pre-compression in the thickness direction, respectively); and (**b**) high temperature stretched sample (together with typical original, pre-heated and typical recovered samples for comparison).

The stress versus strain curves in cyclic uniaxial tension of pre-compressed and pre-stretched samples are plotted in Figure 3. Two pre-compressed samples were compressed in the thickness direction at 160 °C by 9% and 23%, respectively, while the pre-stretched sample was stretched in the length direction by 150% at 160 °C. It was noticed that further compression at 160 °C resulted in severely distorted uneven samples, which were not suitable for further uniaxial tensile test. A closer look reveals that the stress versus strain curves of pre-compressed samples are very close to those of the recovered samples reported in Figure 2c, i.e., still being brittle. However, after high temperature pre-stretching, the sample becomes ductile, with apparent yielding, long strain plateau (>30%) and slight strain hardening (about 10% strain) before fracture. Furthermore, the Young’s modulus of the pre-stretched sample is also increased.

**Figure 4:**
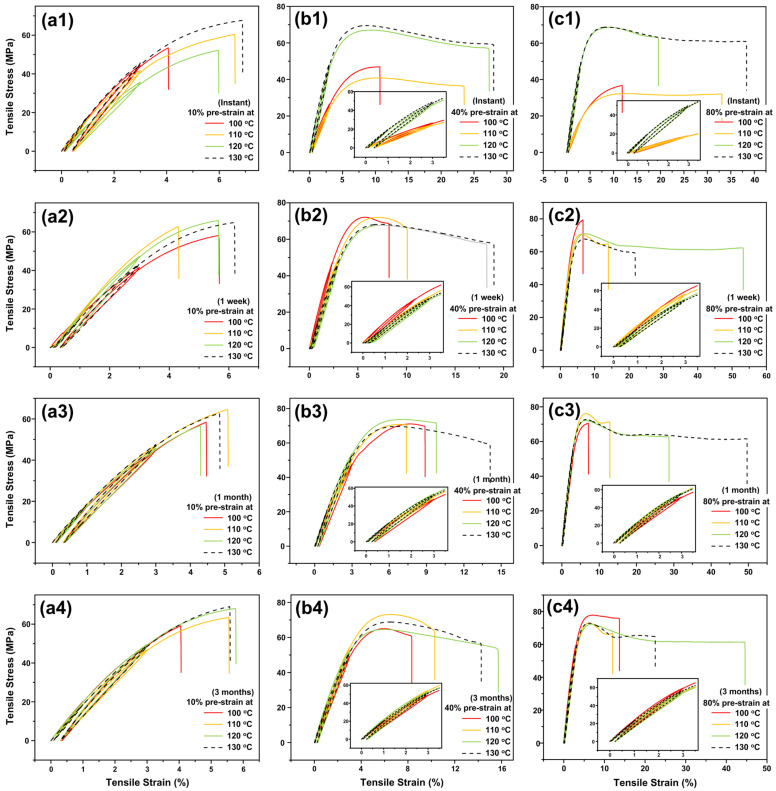
Cyclic uniaxial tension results of high temperature pre-stretched samples (stretched at 100 °C, 110 °C, 120 °C or 130 °C to 10%, 40% or 80% strain, 1 mm Type I samples) after storage at 40 °C for 0 s (instant), one week, one months and three months. (Inset is zoom-in view of the small strain range, and the title and unit of horizontal and vertical coordinates of subfigures are consistent with those of the figure).

Cyclic uniaxial tensile results of high temperature pre-stretched samples (stretched at 100 °C, 110 °C, 120 °C or 130 °C to 10%, 40% or 80% strain using 1 mm Type I sample) after different periods of storage at 40 °C (instant, one week, one month and three months) are replotted in Figure 4. It is clear that depending on the temperature and amount of applied strain in pre-stretching, some samples turn to be ductile.

If the stretching strain is small, e.g., 10% (Figure 4a1–a4), for all four stretching temperatures, the resulted samples are always brittle and the corresponding stress versus strain curves are only slightly different from that of the original sample. Hence, the transition from brittle to ductile is determined by two processing parameters, namely stretching strain and stretching temperature, i.e., the programming strain and programming temperature. Even after three months at 40 °C, the fracture strain of the sample pre-stretched to 80% strain at 120 °C is about 45%. Thus, the transition from brittle to ductile is not a short-term effect. The influence of pre-stretching is not only on the fracture mode, but also on the Young’s modulus (i.e., the slope of the stress versus strain curve in the early loading/unloading part).

### 3.3. XRD

**Figure 5:**
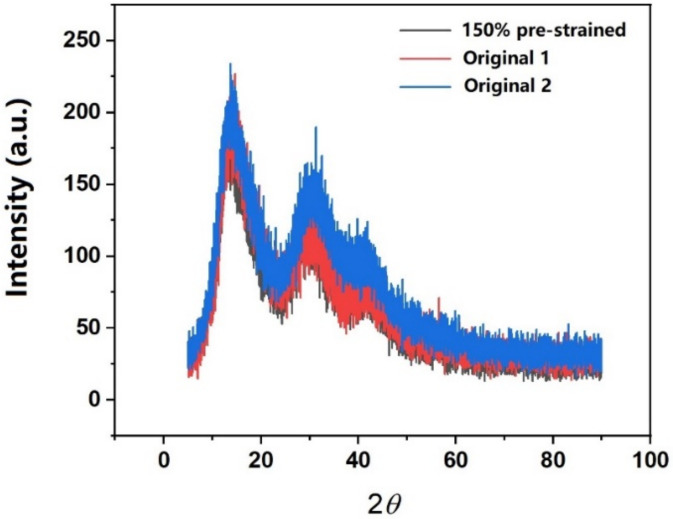
XRD spectra of original samples (two pieces) and 150% pre-stretched (at 160 °C) sample.

Two original samples and one pre-stretched samples (to 150% at 160 °C) were tested. The corresponding XRD spectra are plotted in Figure 5. It appears that the spectrum of the pre-stretched sample is almost the same as those of the original samples. In other words, the microstructure of polymer chains is not changed after high-temperature stretching.

### 3.4. Photoelasticity

**Figure 6:**
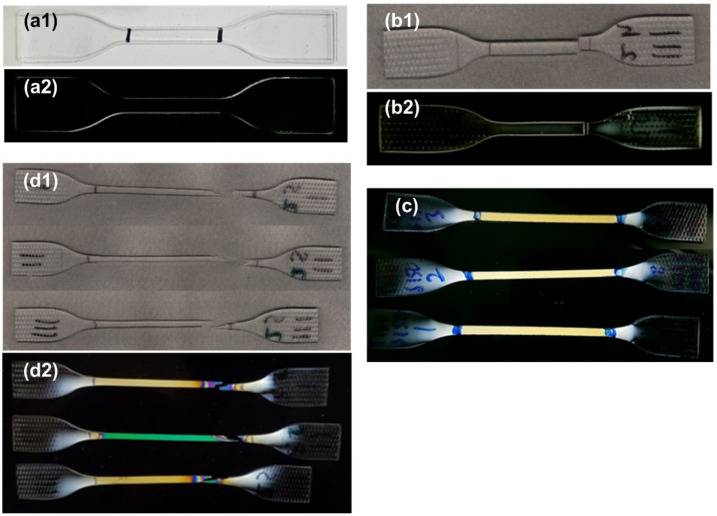
Typical images of (**a1**) original sample (normal optical image); (**a2**) original sample (photoelasticity); (**b1**) typical brittle fracture sample (placed together with a small gap between two fracture parts) (normal optical image); (**b2**) typical brittle fracture sample (placed together with a small gap between two fracture parts) (photoelasticity); (**c**) after 150% stretching at 160 °C (photoelasticity, three pieces); (**d1**) typical ductile fracture samples (normal optical image); and (**d2**) typical ductile fracture samples (photoelasticity).

Figure 6 presents typical photoelasticity images of different samples (2 mm, Type II), i.e., original sample, brittle fracture sample after cyclic uniaxial stretching, sample after high temperature stretching (to 150% at 160 °C) and ductile fracture sample (high temperature pre-stretched). For reference, normal optical photos of some samples are also included.

For the photoelasticity image of a sample, a uniformly colored area indicates a uniform internal elastic stress field, while a rainbow-like colorful area is meant for a gradient internal elastic stress field. As we can see, there is only one uniform color within the gauge length in the original sample (Figure 6a2), brittle fracture sample (Figure 6b2) and pre-stretched sample (Figure 6c). However, in the ductile fracture sample (Figure 6d2), which has been pre-stretched to 150% at 160 °C, the areas around the fracture surfaces are colorful (Figure 6d2). Pre-compressed samples are similar to other brittle fracture samples (refer to Figure S4 in Supplementary Materials).

Recall the corresponding stress versus strain curves in cyclic uniaxial stretching in Figure 2 and Figure 3, the deformation in all brittle fracture samples is mostly elastic, and their fracture occurs in an instant manner. Hence, the corresponding fracture toughness is low, i.e., once there is a crack, the material fractures immediately. Consequently, the corresponding stress intensity factor (SIF) is high. On the other hand, the pre-stretched sample has a much higher toughness, i.e., the area underneath the stress versus strain curve is much larger. A long stress plateau indicates the propagation of the necking front during stretching. The fracture surface, which is not perpendicular to the direction of stretching, implies the possibility of shearing failure, i.e., sliding induced fracture. The rainbow-like colorful areas around the facture surfaces, which are associated with a gradient elastic stress distribution, indicate significant and gradient plastic deformation. Hence, the SIF of pre-stretched samples is low.

At this point, we may conclude that although there is no significant change in structure (recall XRD results in Figure 5), pre-stretching at high temperatures remarkably decreases the SIF of the material, and thus changes the failure mode from brittle to ductile.

Pre-compression in the thickness direction at high temperatures, pre-heating and shape memory cycle via stretching at high temperatures do not affect the SIF, so the fracture mode is still brittle.

In [23], it is mentioned that thick PC samples can undergo brittle failure. According to the classic fracture mechanics (e.g., [43]), the fracture toughness has different values when measured under plane stress and plane strain. After programming via stretching, the thickness of the samples is reduced, which may result in the increase in fracture toughness as the stressing mode may be changed from plane strain to plane stress. Figure S2 (typical results) in Supplementary Materials reveals that there is not much difference in the stress versus strain curves in the samples of two different thicknesses (1 mm and 2 mm). Thus, in the current study, the reduction in sample thickness after stretching is not directly associated with the brittle-ductile transition.

### 3.5. Evolution after Storage at 40 °C

**Figure 7:**
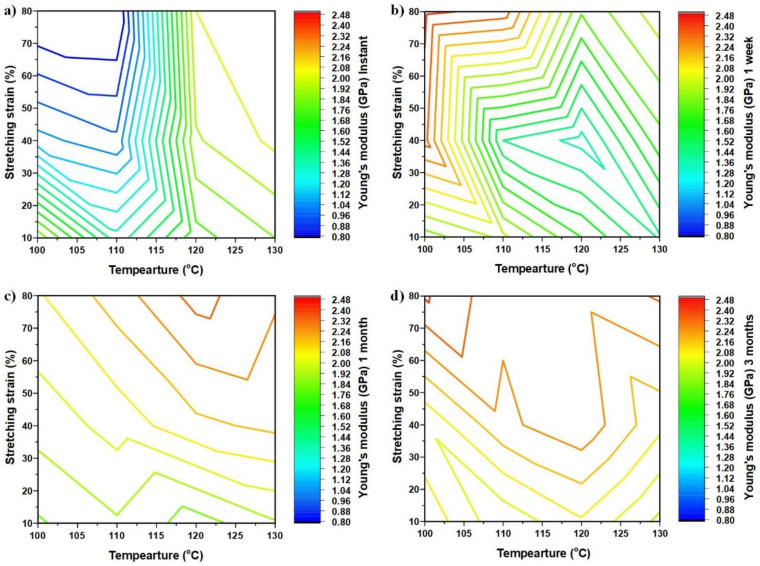
Evolution of the Young’s modulus. (**a**) Instant; (**b**) after one week at 40 °C; (**c**) after one month at 40 °C; (**d**) after three months at 40 °C.

**Figure 8:**
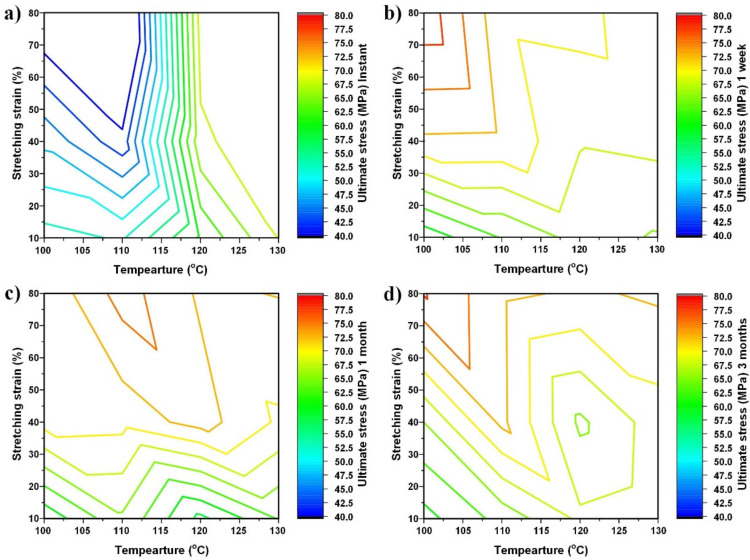
Evolution of ultimate stress. (**a**) Instant; (**b**) after one week at 40 °C; (**c**) after one month at 40 °C; and (**d**) after three months at 40 °C.

**Figure 9:**
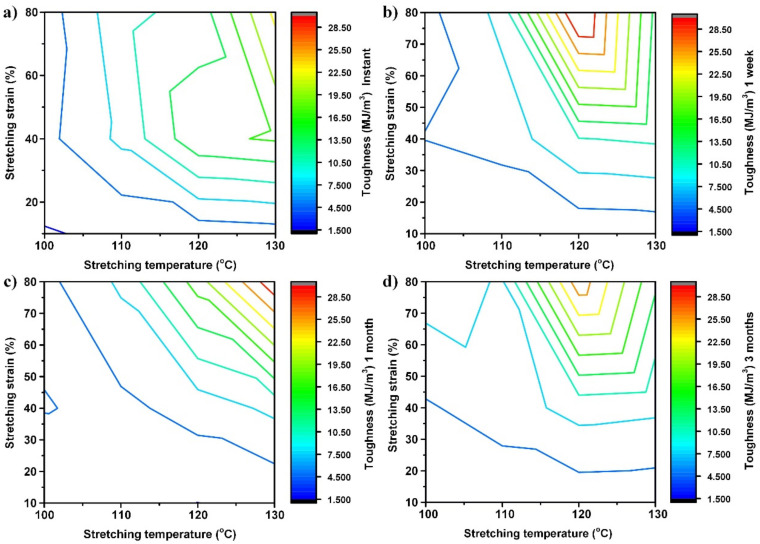
Evolution of toughness. (**a**) Instant; (**b**) after one week at 40 °C; (**c**) after one month at 40 °C; and (**d**) after three months at 40 °C.

The Young’s modulus, ultimate stress (the maximum engineering stress in cyclic stretching to fracture) and toughness of the tested samples are listed in Table S1 in Supplementary Materials. For easier visualization, the contours of the Young’s modulus, ultimate stress and toughness as a function of stretching strain and stretching temperature after storage at 40 °C for different times are plotted together in Figure 7, Figure 8 and Figure 9, respectively, using Matlab’s contour function.

Although the amount of experimental data is limited and the contour may not be very accurate, the general trend revealed here is clear.

From the real engineering application point of view, instant and long-term storage (e.g., after three months) results are more of our interest. Therefore, we will only focus on them in the following discussion.

Figure 7a is the contour of the Young’s modulus right after high-temperature stretching. According to Figure S2 in Supplementary Materials, the Young’s modulus of the original sample (1 mm thick) is about 1.7 GPa. Hence, it is clear that depending on the stretching temperature and stretching strain, the programmed material can become harder or softer. Low temperature stretching reduces the Young’s modulus, while high temperature stretching increases the Young’s modulus.

A closer look reveals that we may roughly split the stretching temperature into three regions, namely: Region I (100 °C to 110 °C, i.e., the glass transition start temperature to T_g_ according to Figure 1a); Region II (110 °C to 120 °C, i.e., the T_g_ to glass transition finish temperature according to Figure 1a); and Region III (120 °C to 130 °C, i.e., above the glass transition finish temperature according to Figure 1a).

Within Region I, with the increase in stretching strain, the Young’s modulus decreases and the influence of the stretching temperature on the Young’s modulus is relatively insignificant when the pre-strain of sample is over 40%. On the contrary, in Region II, the influence of the stretching strain is insignificant, while that of the stretching temperature is obvious. With the increase in stretching temperature, the Young’s modulus increases rapidly. As compared with the changes in Regions I and II, in Region III (above the glass transition temperature range according to Figure 1a), the Young’s modulus increases with the increase in stretching strain and temperature.

It is worth noting that Figure 8a, which is the ultimate stress of the samples without storage, shares almost the same contour pattern as that of the Young’s modulus in Figure 7a. As defined above, here, the ultimate stress is meant for the maximum engineering stress in cyclic stretching to fracture. According to Figure 4a1,b1,c1, except the sample pre-stretched to 80% strain at 110 °C, in which the stress after reaching the peak of yielding is about constant until fracture, the ultimate stresses of all other samples are their fracture stress (for brittle samples) or their yield stress (for ductile samples). Thus, if the real stress and the real strain are used, the ultimate stress mostly corresponds to the fracture stress, regardless of the fracture mode.

A brief summary of the stress verses strain curves of the original sample, pre-heated sample, pre-stretched sample and recovered sample in cyclic uniaxial stretching to fracture in Figure 3b shows that:

(a) Pre-heating does not alter their ultimate stress and the Young’s modulus;

(b) After high-temperature stretching (150% stretched at 160 °C), the Young’s modulus increases, while the ultimate stress is about the same as that of the original sample;

(c) After recovery, both the ultimate stress and Young’s modulus are lower than those of the original sample. The above-mentioned “paracrystalline” [37] might be the reason.

Figure 7a and Figure 8a reveal that the Young’s modulus of the samples stretched at high temperatures to high strains becomes higher, while the corresponding ultimate stress is about the same. This trend is consistent with (b) mentioned above.

In Region I, the stretching temperature is relatively low, and thus the polymer is mostly in the glassy state. The rotation of segments is at a relatively low level, and the polymer chains cannot fully adapt the extension which increases the free volume. Thus, the segments of the polymer become relatively easier to rotate when subsequently stretched at room temperature. Consequently, the Young’s modulus and the ultimate stress decreases continuously with the increase in stretching strain in Region I. The increase in stretching temperature within Region I slightly increases the mobility of polymer segments. Therefore, the Young’s modulus and ultimate stress decrease slightly less with the increase in stretching temperature.

With further increase in stretching temperature, the polymer is in more of a rubbery state, and the chains are easier to align upon stretching. In this study, the samples after stretching were cooled in the air. In Region II, most segments have been activated since the stretching temperature is above the T_g_. The effects of “rejuvenation” are limited [44]. Hence, the influence of stretching strain on the Young’s modulus and ultimate stress in the pre-stretched samples is minimized. The alignment results in the decrease in the free volume and higher density, and consequently increases the Young’s modulus and ultimate stress of the pre-stretched materials.

The Young’s modulus and ultimate stress of the samples pre-strained to around 30% or more, increasing significantly (mostly doubled), with the increase in stretching temperature within Region II. The polymer chains are well-aligned after large extension (e.g., 30% or more).

In Region III, the material is about in full rubbery state during the process of pre-straining. The free volume of the samples pre-strained at high temperatures is relatively smaller after cooling. Thereafter, the mobility of polymer chain is impeded that results in an increase in both the Young’s modulus and ultimate stress.

Not only the contour pattern, but also the magnitude of the Young’s modulus and ultimate stress evolve remarkably after storage, as revealed in Figure 7 and Figure 8, respectively. After three months of storage, the Young’s moduli of all samples increase, all being higher than that of the original sample. About a 40% increment is observed in those samples pre-stretched to more than 40%, at around 117.5 °C. A closer look reveals that there are two peaks for the Young’s modulus, one at (100 °C, 80% pre-strain) and the other at (130 °C, 80% pre-strain). It is interesting to see that there are also two troughs, one at (100 °C, 10% pre-strain) and the other at (130 °C, 10% pre-strain). As a general trend, after physical aging, the more pre-stretched samples have a higher Young’s modulus.

On the other hand, although the increase in the ultimate stress is also observed in all samples after three months of storage, the contour pattern of the ultimate stress after three months of physical aging is slightly different from that of the Young’s modulus after three months of storage. The most apparent difference is that there is a trough at around (120 °C, 40% pre-strain), instead of at (130 °C, 10% strain) in the contour of the Young’s modulus after three months of aging, which results in only one apparent peak at (100 °C, 80% pre-strain).

As mentioned above, the free volume in the samples pre-stretched at low temperatures is relatively larger than that in those samples pre-stretched at high temperatures, as the material is more in the glass state at low temperatures.

A relative larger free volume makes it easier for polymer segments to rotate during physical aging [45]. Without aging, the decrease in the Young’s modulus due to the increase of the pre-strain in Region I (low temperature area) is strongly associated with the free volume in the pre-stretched samples.

During storage (physical aging), the free volume in the pre-stretched samples gradually decreases via relaxation of the polymeric segments. Combined with the alignment of the segments due to pre-stretching, the increase in both the Young’s modulus and ultimate stress is resulted.

Figure 9 clearly reveals that although pre-stretching does enhance the toughness of the material, the combination of high stretching temperature and high stretching strain is more effective. Refer to Table S1 in Supplementary Materials for the original experimental results. For instantly tested samples as shown in Figure 9a, the toughness of the sample stretched at 100 °C to 10% strain is 1.317 MJ/m^3^, while that of the sample stretched at 130 °C to 80% is 23.281 MJ/m^3^, increased over 16 times. After aging for three months, the contour pattern of toughness (Figure 9d) is slightly different. The maximum toughness is found in the sample pre-stretched at 120 °C to 80% strain, and the variation of toughness ranges from 1.476 MJ/m^3^ to 27.532 MJ/m^3^. It appears to be feasible to significantly tailor the toughness of this PMMA via high-temperature programming.

To achieve the combination of high Young’s modulus and high toughness, a high stretching strain at the glass transition finish temperature appears to be the most effective manner.

## 4. Mechanism of Brittle-Ductile Transition

According to [23], there are two kinds of interaction within amorphous polymers: one is a strong molecular chain network and the other is weak intermolecular interaction. The brittle fracture in amorphous polymers is due to cracking, while the ductile fracture is the result of shearing.

**Figure 10:**
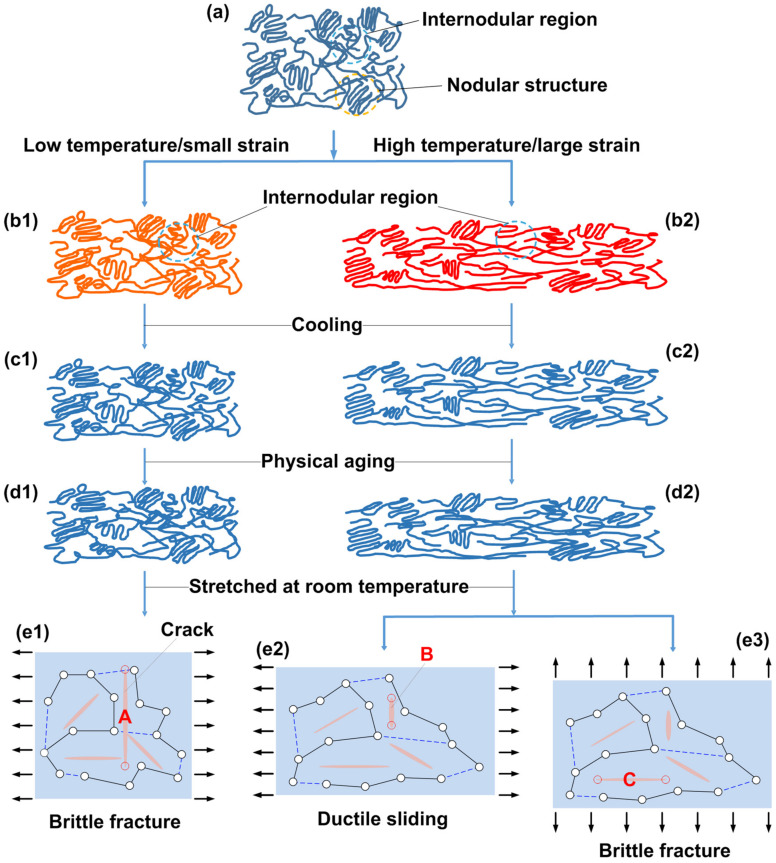
Schematic illustration of the mechanism of brittle-ductile transition after high temperature programming/aging. In (**e1**–**e3**), solid black line: molecular chain network; dashed blue line: intermolecular interaction [23].

Together with the experimental results reported above, the possible mechanism of brittle-ductile transition after high temperature programming via uniaxial stretching and then aging can be schematically sketched, as illustrated in Figure 10. For simplicity, we only consider two typical types of programming: programming at low temperatures (around the glass transition start temperature) to small strain; and programming at high temperatures (around the glass transition finish temperature) to large strain. After programming and aging, the material is stretched to fracture either along the direction of previous programming or along the direction which is perpendicular to the previous programming direction. From the mechanism point of view, the latter is similar to the cases of pre-compressed samples (Figure 3a, in the thickness direction), since the direction of programming is perpendicular to the direction of the second stretching to fracture.

In the original glass state Figure 10a, nodular structures are connected by internodular regions. There are many tine cracks (micro-gaps). Upon stretching (programming),

- If the temperature is low (Figure 10b1, the polymer is still relatively rigid) and the strain is small, the nodular structures are largely intact, while the internodular regions deform slightly to accommodate the required deformation. The tine cracks within the polymer mostly remain (similar to Figure 10e1).
- If the temperature is high (Figure 10b2, the polymer is much softer) and the strain is large, both the nodular structures and internodular regions deform remarkably to match the high strain requirement. During stretching, the molecular chains re-align and the cracks reshape (rotation, elongation in the stretching direction and shrinkage in the transverse direction of stretching) (similar to Figure 10e2,e3). Sliding is in favor to enable large stretching at high temperatures, and the polymer turns to be more packed. Given a polymer, there is always a stretching limit. Overstretching not only weakens the polymer network, but also limits the capability of the pre-stretched polymer to stretch further.

After constrained cooling, the freestanding sample is again in the glassy state, and the deformation and micro-structure are mostly maintained. There is not any change in bonding, but depending on the programming temperature and programming strain, the microstructure of the polymer may be remarkably different (Figure 10c1,c2). Long-term aging enables relaxation/creeping to reduce the internal stress. Hence, the microstructure and even the overall shape of the sample may slightly change (Figure 10d1,d2). The changes are more apparent if the pre-stretching strain is high while the pre-stretching temperature is low, since more free volume is produced in these samples after programming. After aging, the free volume will be reduced and the sample becomes dense.

Due to the difference in microstructure after programming, the sample programmed at low temperature to small strains is easier to fracture upon stretching along the direction of programming, since the original cracks (e.g., A in Figure 10e1), which cause brittle fracture of the original sample, are still there. If the applied programming is larger, the Young’s modulus of the programmed sample decreases, due to the widening of the cracks; meanwhile, the toughness does not change much, since toughness is more relevant to SIF, which does not change too much.

For the sample programmed at high temperatures to high strains, if stretching follows the same direction as that applied in programming (Figure 10e2), the sliding system established during programing enables the sample for further high extension before fracture. The crack (B) turns to be less influential, due to high-temperature/large strain programming. The corresponding Young’s modulus increases as the polymer is more packed after programming. If the applied programming strain is small, the cracks mostly remain, and no sliding system is activated. Consequently, the polymer is still brittle and the Young’s modulus is about the same. If the stretching direction is perpendicular to the programming direction (Figure 10e3), the material is still brittle, as the sliding system only works in a vertical direction, which is perpendicular to the direction of stretching. The failure is controlled by crack C.

Figure S5 (Supplementary Materials) presents some typical optical microscopic images of the samples at different stages. Apart from some small particles, there is not any remarkable feature in the original sample (Figure S5a). After stretching to 150% at 160 °C, parallel lines, which look like sliding [46], can be observed (Figure S5b). Figure S5c is the typical whole cross-section (top view) of a brittle fracture sample. A closer look (Figure S5d from various areas, not limited to Figure S5c) reveals many seemingly regular patterns, which look similar to the typical features of dislocation [46], although this material is an amorphous polymer and the scale of these features is on the order of micrometers. Further investigation is required to identify what these patterns exactly are.

## 5. Conclusions

PMMA, a typical brittle amorphous polymer with excellent SME, is investigated to reveal the influence of stretching strain, stretching temperature and aging on its toughness (brittle-ductile transition), Young’s modulus and ultimate stress (maximum engineering stress in uniaxial tension to fracture).

It is observed that the toughness of this PMMA can be tailored via controlling the temperature and strain in pre-stretching. The combination of high temperature and high strain results in a remarkable increase in toughness (ranging from 1.317 MJ/m^3^ to 23.281 MJ/m^3^). After aging at 40 °C for three months, the toughness changes slightly (ranging from 1.476 MJ/m^3^ to 27.532 MJ/m^3^).

The Young’s modulus of pre-stretched PMMA without aging may decrease or increase depending on the combination of stretching temperature and stretching strain. At low temperatures, higher stretching strain reduces the Young’s modulus, while at high temperatures, the Young’s modulus is higher upon stretching. If the stretching temperature is between the T_g_ and the glass transition finish temperature, the Young’s modulus of the stretched sample is highly dependent on the stretching temperature. A higher stretching temperature results in a higher Young’s modulus. The influence of stretching strain is only limited to the small stretching strain range. After three months of aging, the Young’s moduli of all samples are higher than that of the original sample.

The influence of stretching temperature, stretching strain and aging on the ultimate stress follows about the same trend as that of the Young’s modulus. If the real stress is applied, the ultimate stress of almost all samples is associated with their fracture stress, regardless the actual fracture mode.

To achieve the combination of high Young’s modulus and high toughness, a high stretching strain at the glass transition finish temperature appears to be the most effective approach.

Together with the results of a series of additional experiments, which include DSC, XRD and photo-elasticity, etc., on preheated, shape recovered, pre-compressed and pre-stretched samples, a modified mechanism for the brittle-ductile transition via high temperature (around T_g_) stretching is proposed. High strain stretching at high temperatures builds in a sliding system within the material, which helps for further stretching along the direction of the previous pre-stretching/programming.

## Data Availability

Not applicable.

## Supplementary Materials

Figure S1: Samples of (a) Type I and (b) Type II. (Unit: mm); Figure S2 Comparison of stress versus strain curves of 1 mm (black) and 2 mm (red) thick samples (original, type II) in cyclic uniaxial tension; Figure S3 Comparison of stress versus strain curves of 1 mm thick original (blue) and recovered (orange) samples (type II) in cyclic uniaxial tension; Figure S4 Pre-compressed samples. (a) Pre-compressed to 23% at 160 °C in the thickness direction; (b) Pre-compressed to 9% at 160 °C in the thickness direction. (1) After pre-compression (photoelasticity); (2) after cyclic uniaxial tension to fracture (photoelasticity); (3) after cyclic uniaxial tension to fracture (normal photo); Figure S5 Typical microscopic images. (a) Original sample; (b) after stretching (in horizontal direction) to 150% at 160 °C; (c) typical cross-section of brittle fracture sample; (d1–d4) typical features observed in brittle fracture samples (stretched in the vertical direction). Unless otherwise specified, the scale bar is 100 μm; Table S1: Young’s modulus (a), ultimate stress (b) and toughness (c).
