# Supplementary material for: On-Demand Tailoring between Brittle and Ductile of Poly(methyl methacrylate) (PMMA) via High Temperature Stretching
Source: Polymers (Basel). 2022 Feb 28;14(5):985. doi: 10.3390/polym14050985 (PMC8912607; doi:10.3390/polym14050985)
Supplement: Supplementary file 1 [file polymers-14-00985-s001.zip › polymers-1569566-supplementary.pdf]

Supplementary

On-demand tailoring between brittle and ductile of poly(methyl methacrylate) (PMMA) via high temperature stretching

Changchun Wang[1,*] , Jia Xi Pek[2], Hong Mei Chen[3] and Wei Min Huang[2,*]

1 Jiangsu Key Laboratory of Advanced Structural Materials & Application Technology, School of Material Science and Engineering, Nanjing Institute of Technology, Nanjing 211167, China

2 School of Mechanical and Aerospace Engineering, Nanyang Technological University, 50 Nanyang Avenue, Singapore 639798, Singapore

3 College of Chemistry and Materials Science, Sichuan Normal University, Chengdu 610066, China

* Correspondence: ccwang@njit.edu.cn and mwmhuang@ntu.edu.sg

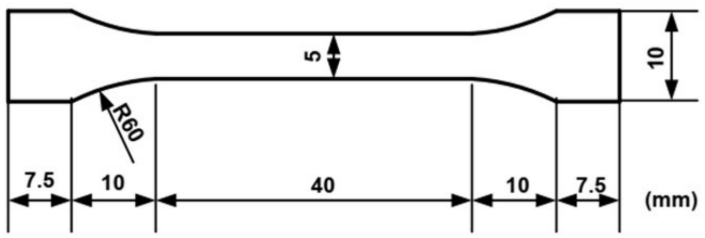

(a)

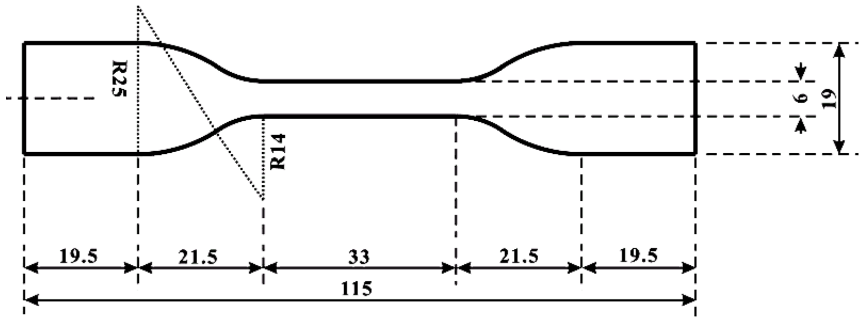

(b)

Figure S1 Samples of (a) Type I and (b) Type II. (Unit: mm)

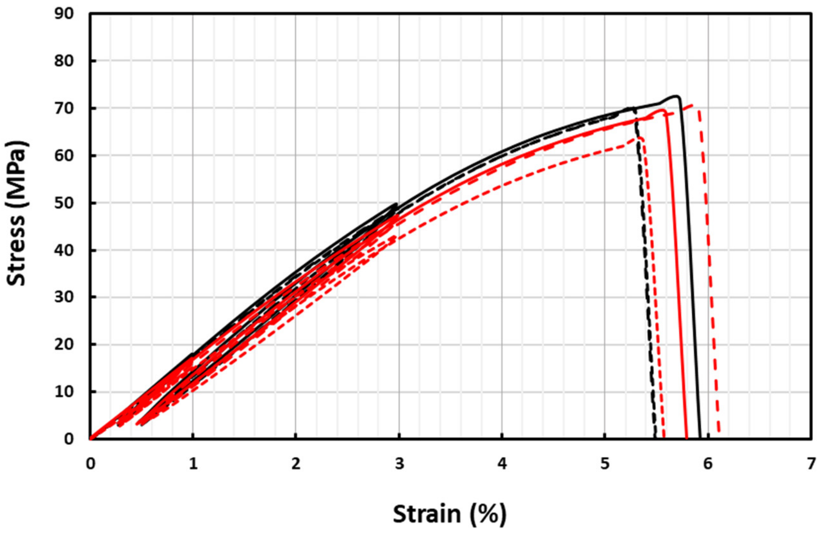

Figure S2 Comparison of stress versus strain curves of 1 mm (black) and 2 mm (red) thick samples (original, type II) in cyclic uniaxial tension.

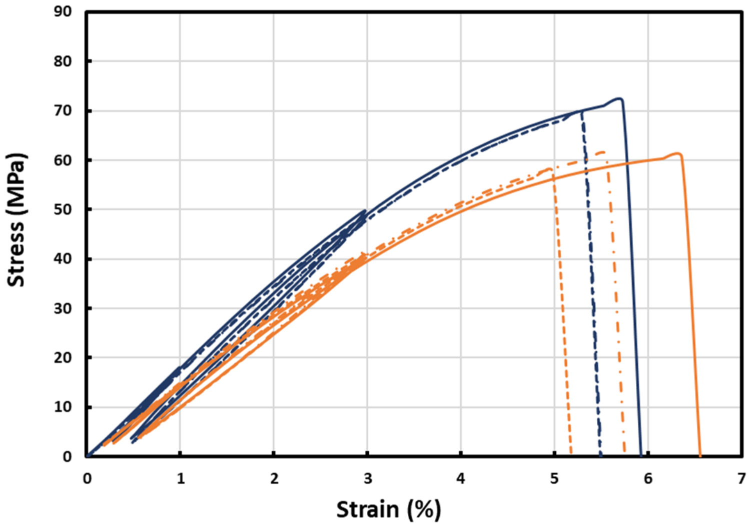

Figure S3 Comparison of stress versus strain curves of 1 mm thick original (blue) and recovered (orange) samples (type II) in cyclic uniaxial tension.

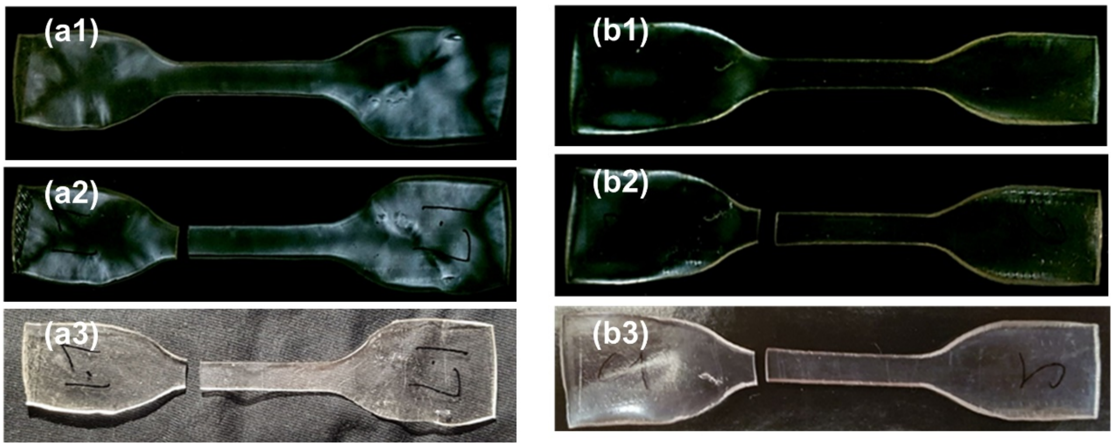

Figure S4 Pre-compressed samples. (a) Pre-compressed to 23% at 160 ºC in the thickness direction; (b) Pre-compressed to 9% at 160 ºC in the thickness direction. (1) After pre-compression (photoelasticity); (2) after cyclic uniaxial tension to fracture (photoelasticity); (3) after cyclic uniaxial tension to fracture (normal photo).

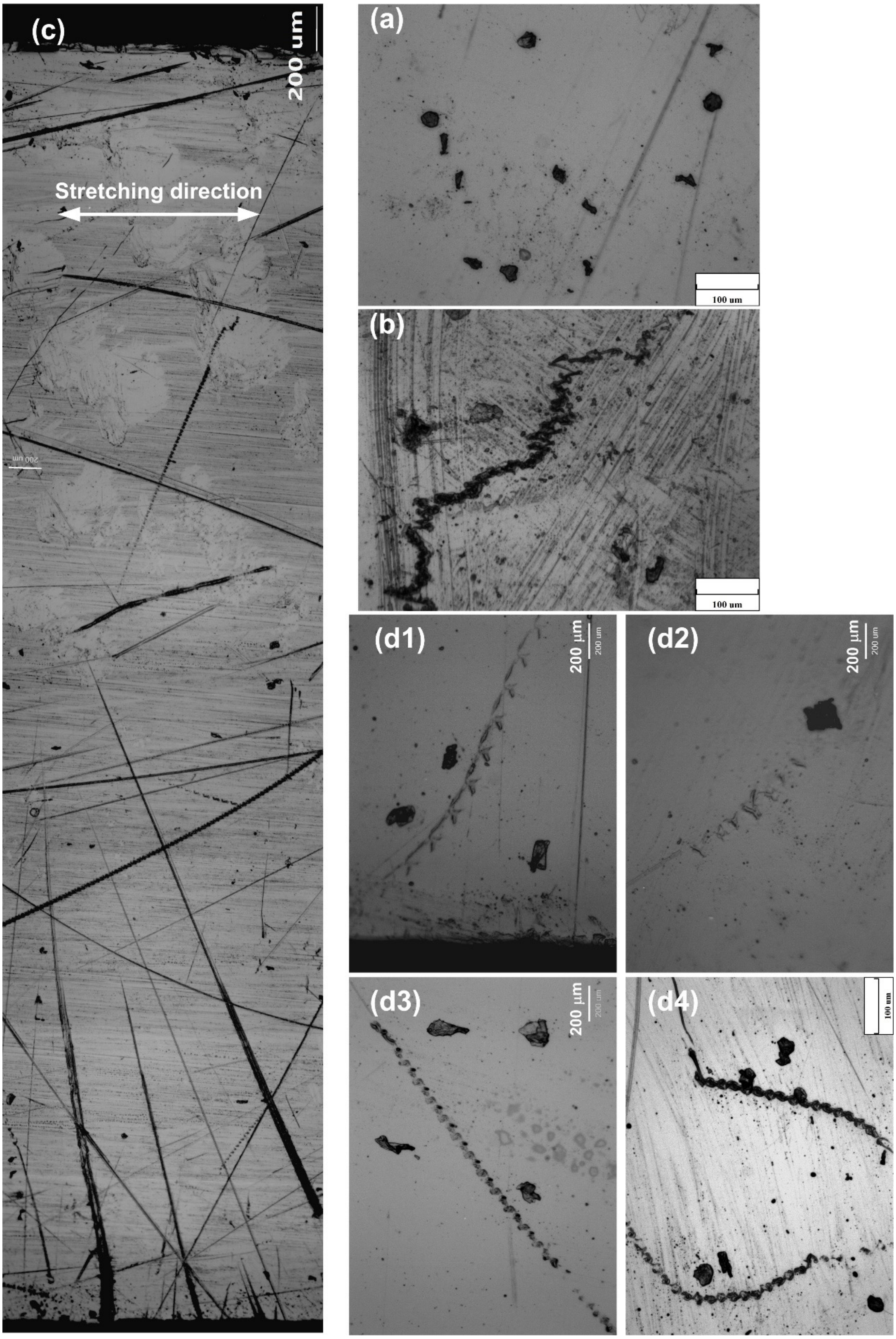

Figure S5 Typical microscopic images. (a) Original sample; (b) after stretching (in horizontal direction) to 150% at 160 ºC; (c) typical cross-section of brittle fracture sample; (d1-d4) typical features observed in brittle fracture samples (stretched in the vertical direction). Unless otherwise specified, the scale bar is 100 μm.

Table S1 Young's modulus (a), ultimate stress (b) and toughness (c).

(a) Young's modulus (GPa)

**Pre-stretching temperature (^oC)**	**Pre-stretching strain (%)**	**Storage time**			
		**Instant**	**1 week**	**1 month**	**3 months**
100	10	1.9	1.8	1.83	2.14
	40	1.07	2.35	1.95	2.01
	80	0.7	2.42	2.07	2.41
110	10	1.39	1.94	1.91	1.94
	40	0.98	1.44	2.03	2.22
	80	0.69	2.37	2.2	2.26
120	10	1.73	1.68	1.8	2.07
	40	1.91	1.33	2.14	2.3
	80	2	1.77	2.35	2.26
130	10	1.84	1.43	1.91	1.9
	40	2.02	1.73	2.18	2.1
	80	2.03	1.97	2.25	2.33

(b) Ultimate stress (MPa)

**Pre-stretching temperature (^oC)**	**Pre-stretching strain (%)**	**Storage time**			
		**Instant**	**1 week**	**1 month**	**3 months**
100	10	53.5	58.4	58.1	59.1
	40	46.9	72.1	71	65
	80	36.8	79.3	70.4	77.7
110	10	52.2	62.7	64.6	63.4
	40	40.8	72	70.8	73.1
	80	32.2	70.6	76.1	73
120	10	60.4	65.9	56.8	68
	40	67	67.7	73.6	64.5
	80	68.7	71	72.1	72.1
130	10	67.6	64.8	62.7	69
	40	69.5	68.2	69.6	68.8
	80	68.8	67.7	72.6	72.9

(c) Toughness (MJ/m^3)

Pre-stretching temperature (^{o}C)	Pre-stretching strain (%)	Storage time			
		Instant	1 week	1 month	3 months
100	10	1.317	2.108	1.564	1.476
	40	3.628	4.557	4.653	4.148
	80	3.004	3.607	3.61	9.14
110	10	1.975	1.534	2.035	2.302
	40	8.178	5.629	3.765	5.985
	80	9.563	8.36	8.043	7.182
120	10	2.659	2.369	1.485	2.588
	40	15.854	10.363	5.708	8.621
	80	11.677	32.751	17.912	27.532
130	10	3.133	2.554	1.884	2.437
	40	16.828	10.952	8.197	8.102
	80	23.281	12.727	30.925	14.064
